# Nipbl and Mediator Cooperatively Regulate Gene Expression to Control Limb Development

**DOI:** 10.1371/journal.pgen.1004671

**Published:** 2014-09-25

**Authors:** Akihiko Muto, Shingo Ikeda, Martha E. Lopez-Burks, Yutaka Kikuchi, Anne L. Calof, Arthur D. Lander, Thomas F. Schilling

**Affiliations:** 1 Department of Developmental & Cell Biology, University of California, Irvine, Irvine, California, United States of America; 2 Center for Complex Biological Systems, University of California, Irvine, Irvine California; 3 Department of Biological Science, Graduate School of Science, Hiroshima University, Higashi-Hiroshima, Hiroshima, Japan; 4 Department of Anatomy & Neurobiology, University of California, Irvine, Irvine, California, United States of America; Stanford University School of Medicine, United States of America

## Abstract

Haploinsufficiency for Nipbl, a cohesin loading protein, causes Cornelia de Lange Syndrome (CdLS), the most common “cohesinopathy”. It has been proposed that the effects of Nipbl-haploinsufficiency result from disruption of long-range communication between DNA elements. Here we use zebrafish and mouse models of CdLS to examine how transcriptional changes caused by Nipbl deficiency give rise to limb defects, a common condition in individuals with CdLS. In the zebrafish pectoral fin (forelimb), knockdown of Nipbl expression led to size reductions and patterning defects that were preceded by dysregulated expression of key early limb development genes, including *fgfs*, *shha*, *hand2* and multiple *hox* genes. In limb buds of *Nipbl*-haploinsufficient mice, transcriptome analysis revealed many similar gene expression changes, as well as altered expression of additional classes of genes that play roles in limb development. In both species, the pattern of dysregulation of *hox*-gene expression depended on genomic location within the *Hox* clusters. In view of studies suggesting that Nipbl colocalizes with the mediator complex, which facilitates enhancer-promoter communication, we also examined zebrafish deficient for the Med12 Mediator subunit, and found they resembled Nipbl-deficient fish in both morphology and gene expression. Moreover, combined partial reduction of both Nipbl and Med12 had a strongly synergistic effect, consistent with both molecules acting in a common pathway. In addition, three-dimensional fluorescent in situ hybridization revealed that Nipbl and Med12 are required to bring regions containing long-range enhancers into close proximity with the zebrafish *hoxda* cluster. These data demonstrate a crucial role for Nipbl in limb development, and support the view that its actions on multiple gene pathways result from its influence, together with Mediator, on regulation of long-range chromosomal interactions.

## Author Summary

Limb malformations are a striking feature of Cornelia de Lange Syndrome (CdLS), a multi-system birth defects disorder most commonly caused by haploinsufficiency for *NIPBL*. In addition to its role as a cohesin-loading factor, Nipbl also regulates gene expression, but how partial Nipbl deficiency causes limb defects is unknown. Using zebrafish and mouse models, we show that expression of multiple key regulators of early limb development, including *shha*, *hand2* and *hox* genes, are sensitive to Nipbl deficiency. Furthermore, we find morphological and gene expression abnormalities similar to those of Nipbl-deficient zebrafish in the limb buds of zebrafish deficient for the Med12 subunit of Mediator—a protein complex that mediates physical interactions between enhancers and promoters—and genetic interaction studies support the view that Mediator and Nipbl act together. Strikingly, depletion of either Nipbl or Med12 leads to characteristic changes in *hox* gene expression that reflect the locations of genes within their chromosomal clusters, as well as to disruption of large-scale chromosome organization around the *hoxda* cluster, consistent with impairment of long-range enhancer-promoter interaction. Together, these findings provide insights into both the etiology of limb defects in CdLS, and the mechanisms by which Nipbl and Mediator influence gene expression.

## Introduction

Cohesin, a ring-shaped, DNA-associated protein complex, is best known for its role in tethering sister chromatids together until mitosis [1], [2]. However, growing evidence indicates that cohesin, and proteins such as Nipped-B-like (Nipbl) that regulate cohesin loading onto DNA, also play critical roles in gene regulation [3]–[13]. In particular, it has been suggested that Nipbl and cohesin mediate interactions between promoters and distant enhancers, a process thought to involve the physical looping out of intervening DNA sequences [14]–[16]. For example, in *Drosophila*, Nipped-B (the orthologue of Nipbl) and cohesin regulate *cut* gene expression by controlling long-range interactions between the *cut* promoter and a wing-specific remote enhancer [5]. In mice, haploinsufficiency for *Nipbl* impairs looping that controls the selective expression of beta-globin isoforms by erythroid cells [13].

Recently, it was found that Nipbl co-localizes with the Mediator complex at promoters/enhancers of actively transcribed genes in mouse embryonic stem cells [17]. Thought to play a pivotal role in transmitting regulatory signals from gene-specific activators/repressors to RNA polymerase II [18], [19], Mediator is a large complex composed of a core that interacts with RNA polymerase II and gene-specific transcriptional regulators, and a Cdk8 submodule (containing Cdk8, CyclinC, Med12 and Med13) and can either negatively [20]–[22] or positively [23], [24] regulate transcription. The reported physical interaction between Mediator and Nipbl at active genes suggests that they function together in promoter-enhancer communication, but exactly how this occurs is unknown.

Much insight into the physiological significance of cohesin's influence on transcription has come from the study of Cornelia de Lange Syndrome (CdLS) and other “cohesinopathies”. CdLS is a congenital syndrome characterized by growth retardation, neurological dysfunction, and structural defects in multiple organs [25]–[30], and is caused, in most cases, by haploinsufficiency for *NIPBL* [31], [32]. More recently it has been shown that mutations in cohesin subunits SMC1A or SMC3 [33], [34] or the SMC3 deacetylase, HDAC8 [35], are less common causes of CdLS. Analysis of both patient samples and animal models indicate that *Nipbl* haploinsufficiency causes small changes (usually less than 1.5-fold) in the expression of many hundreds of genes [3], [4], [11]. Analysis of both mouse and fish models of Nipbl deficiency suggests that pervasive phenotypic abnormalities result from the collective, and sometimes synergistic, effects of such small changes in gene expression [3], [11].

Among the most striking abnormalities in CdLS are limb defects, which range from mild brachydactyly and clinodactyly to severe digit and limb truncations, the latter in about 1/3 of cases [26], [28], [36]. Limb reduction is one of the few structural defects in CdLS that is not replicated in the *Nipbl*-haploinsufficient mouse model, as these mice exhibit only minor changes in the shape of the olecranon process, and delays in the ossification of limb bones [3]. Hypothesizing that this difference might reflect slight differences in the threshold for triggering such defects in mouse versus man, we decided to look at development of the pectoral fin (the homologue of the mammalian forelimb) in a zebrafish model of Nipbl-deficiency, produced by injection of morpholino oligonucleotides (MO) directed against the two zebrafish *nipbls* [11]. Here we show that Nipbl-deficient fish display a marked reduction in pectoral fin size, which is already apparent early in fin bud development. We demonstrate that Nipbl is required for normal expression of conserved regulators of vertebrate limb growth and patterning, including *fgfs* in the apical ectodermal ridge (AER), *shh* in the zone of polarizing activity (ZPA), and several *hox* genes of the *hoxab*, *hoxca* and *hoxda* clusters. We also show that that *Nipbl*-haploinsufficient mouse limb buds display a pattern of gene expression changes strikingly similar to those observed in Nipbl-deficient pectoral fin buds.

Pectoral fin defects have also been reported in *med12*-mutant zebrafish, in which Mediator function is disrupted [37]. Interestingly, we find that both the morphological and gene expression changes that occur in Nipbl-deficient fin buds are mimicked when *med12* is knocked down. In particular, expression of multiple *hox* genes in different clusters is affected in a similar position-specific manner in both Nipbl- and Med12-deficient fish embryos, and results of experiments in which we simultaneously knock down both Nipbl and Med12 suggest that they interact genetically. Using 3-dimensional fluorescent in situ hybridization (3D-FISH) in zebrafish fin buds, we further show that Nipbls and Med12 are required for higher-order chromatin organization near the *hoxda* cluster. Overall, the data point to a shared, conserved role for Nipbl and the Mediator complex in the regulation of long-range enhancer-promoter interactions underlying growth and patterning of the vertebrate limb.

## Results

### Impaired pectoral fin development in Nipbl-deficient zebrafish

**Figure 1:**
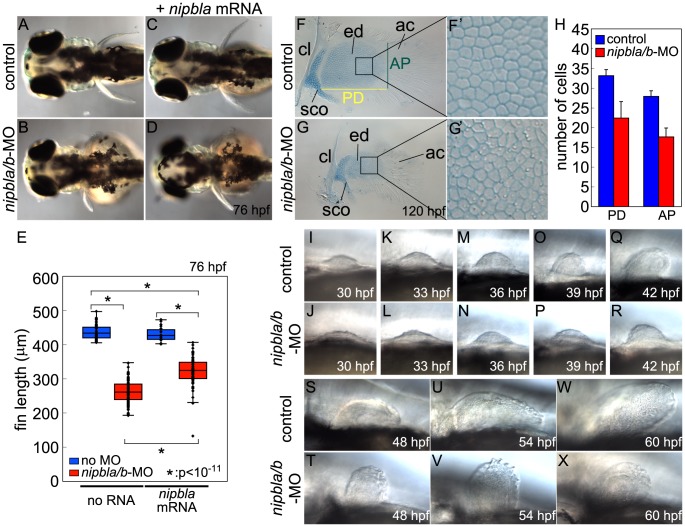
Nipbl knockdown disrupts pectoral fin development. (A–D) Reduced pectoral fins in live Nipbl-deficient embryos at 76 hpf. Dorsal views, anterior to the left. Uninjected control (A), Nipbl-deficient (*nipbla/b*-MO) (B), injected with 400 pg of *nipbla* mRNA alone (C) and co-injected with *nipbla/b*-MO+*nipbla* mRNA (D). (E) Whisker plots of fin length at 76 hpf; medians: 431.8 µm, n = 50 (control), 258.5 µm, n = 88 (*nipbla/b*-MOs), 423.0 µm, n = 22 (*nipbla* mRNA alone), and 319.5 µm, n = 70 (*nipbla/b*-MOs+*nipbla* mRNA). p-values are indicated in the graph. (F, F′, G, G′) Alcian blue staining of cartilages in pectoral fins in control (F, F′) and Nipbl-deficient embryos (G, G′) at 120 hpf. F′ and G′ are higher magnification pictures of boxed areas of endoskeletal discs in F and G, respectively. ac, actinotrichs; cl, cleithrum; ed, endoskeletal disc; sco, scapulocoracoid. (H) Numbers of endoskeletal cells in pectoral fins along proximodistal (PD) and anteroposterior (AP) axes (control; n = 13, Nipbl-deficient embryos; n = 16). PD (Ave ± S.D.): 33.2±1.5 (control) and 22.4±4.2 (Nipbl-deficient embryos), p<10^−8^. AP (Ave ± S.D.): 27.9±1.5 (control) and 17.7±2.2 (Nipbl-deficient embryos), p<10^−13^. (I-X) Morphology of developing pectoral fin buds in live embryos. Lateral views, anterior and dorsal to the left and top, respectively.

Both *nipbl* genes in zebrafish, *nipbla* and *nipblb* [11], are expressed in developing pectoral fin bud mesenchyme (Fig. S1A, B). To investigate their requirements in forelimb development, we generated “Nipbl-deficient” embryos in which both *nipbla* and *nipblb* were depleted by injecting either of two different sets of antisense morpholinos (MOs) designed against distinct MO target sites, as described previously [11]. Pectoral fins of Nipbl-deficient larvae were 40% shorter in length at 72 hours post fertilization (hpf) than those of control embryos (Figure 1A, B, E). This reduced size was not simply due to developmental delay, since it was much more severe than expected given the delay in development of the eye and lower jaw, which we estimated at approximately 16 hrs (Figure S2, see below). Pectoral fin defects were more severe when MOs to both *nipbl* mRNAs were injected, compared with either one alone (Figure S3) [11]; thus, injections of both MOs were used in subsequent experiments. Pectoral fin defects were partially rescued by co-injection of exogenous *nipbla* mRNA, confirming MO specificity (Figure 1C–E).

Alcian Blue staining at 5 dpf revealed that pectoral fin cartilages of the endoskeletal discs form in Nipbl-deficient larvae but are smaller (Figure 1F, G). Cell numbers in these discs were reduced by 37% and 33% along anterior-posterior (A-P) and proximo-distal (P-D) axes, respectively (Figure 1H), whereas cell size resembled controls (Figure 1F′, G′) and we found no change in cell death (Figure S4A, B), suggesting impaired growth of cartilage progenitors at earlier stages. In addition, the orderly arrangement and spacing of chondrocytes in the endoskeletal disc cells was noticeably disrupted in Nipbl-deficient fins (Figure 1F′, G′).

In zebrafish embryos, pectoral fin buds first appear at 30 hpf as shallow domes along the A-P axis, and grow and begin to fold posteriorly by 42 hpf. In Nipbl-deficient embryos, pectoral fin buds also initiate at 30 hpf but grow more slowly than controls (Figure 1I–R). TUNEL assays (Figure S4C–E) showed no increase in cell death in Nipbl-deficient fin buds (Figure S4A–E). In contrast, numbers of BrdU+ cells decreased significantly in the mesenchyme of Nipbl-deficient pectoral fin buds (Figure S4F–I). These data suggest that endoskeletal disc size reduction in Nipbl-deficient limb buds reflects cumulative effects of slower rates of cell division.

Since Nipbl is required for embryonic growth in both fish and mice [3], [11], we stage-matched embryos using an independent criterion – i.e. the A-P position of the migrating posterior lateral line (pLL) primordium labeled by in situ hybridization (ISH) for *fgf10a*; Figure S5A, red arrows). In controls, pLL primordia lie just posterior to the pectoral fin buds at 22 hpf, and continue to migrate posteriorly. Based on this staging criterion the developmental delay in Nipbl-deficient embryos (summarized in Figure S5B) cannot account for the severe limb reductions in Nipbl-deficient larvae (Figure 1S–X).

### Nipbls are required for *fgf* expression in the AER but not in fin bud mesenchyme

**Figure 2:**
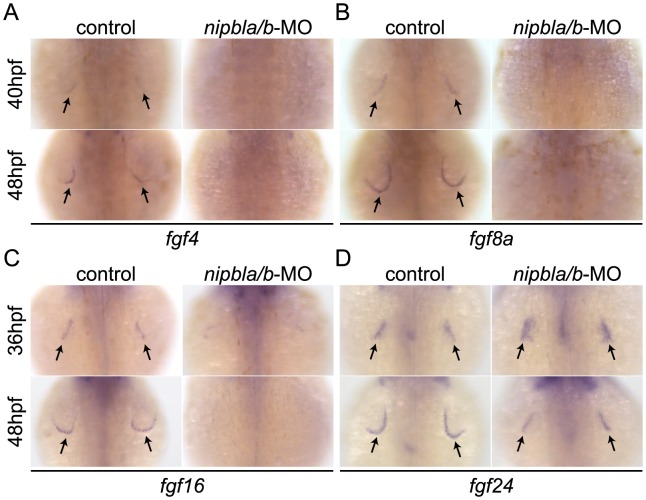
Reduced expression of *fgf*s in the AER of Nipbl-deficient embryos. Expression of *fgf4* (A), *fgf8a* (B), *fgf16* (C) and *fgf24* (D) in the AER (arrows) at indicated stages in control and Nipbl-deficient embryos examined by ISH. Dorsal views, anterior to the top.

Early limb development is highly conserved from fish to mammals [38]–[40]. Each fin/limb bud possesses an apical ectodermal ridge (AER) and zone of polarizing activity (ZPA) [40], [41], which play important roles in growth and patterning [42]–[44]. The AER, a thickened epithelium that rims the distal ends of the buds, is the source of Fgf signals required for P-D limb outgrowth [45]–[48]. The zebrafish AER expresses 4 *fgf* genes: *fgf4*, *fgf8a*, *fgf16* and *fgf24* [49], [50]. Of these, expression of *fgf4*, *fgf8a* and *fgf16* was dramatically reduced in pectoral fin buds of Nipbl-deficient embryos (Figure 2A–C), which was rescued by over-expression of full-length *nipbla* mRNA (Figure S6). This was not simply due to loss of AER cells since *fgf24* expression was not downregulated in the Nipbl-deficient AER (Figure 2D).

Limb bud development also requires expression of *fgf10a* and *fgf24* in the mesenchyme [49], [51], [52]. However, we found no differences in *fgf10a* expression between wild type and Nipbl-deficient limb buds between 22–48 hpf (Figure S7A), as well as no differences in expression of *tbx5a* and *fgf24*, which control the expression of *fgf10a* (Figure S7B–C).

### Nipbls are required for *shh* expression in the ZPA and its regulation in fin bud mesenchyme

**Figure 3:**
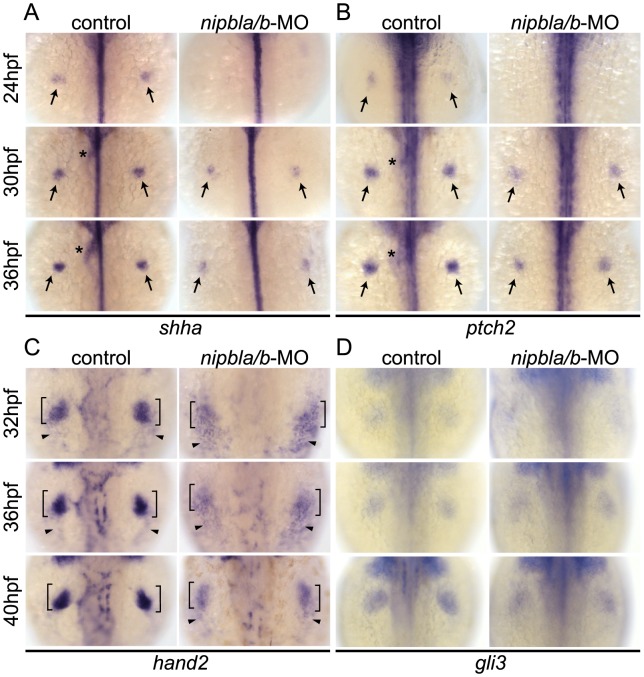
Reduction of genes involved in the *shh*-related gene regulatory cassette in developing pectoral fin mesenchyme of Nipbl-deficient embryos. Expression of fin mesenchymal genes at indicated stages in control and Nipbl-deficient embryos examined by ISH. Dorsal views, anterior to the top. (A, B) Expression of *shha* and *ptch2* in pectoral fin buds (arrows) was significantly reduced in Nipbl-deficient embryos, while midline, neural tube expression was unaffected. Expression in endoderm derived-tissues (asterisks) is also reduced. (C) Expression of *hand2* was also significantly reduced in stage-matched pectoral fin buds. *hand2* expression in the fin buds and posterior lateral plate mesoderm is marked by brackets and arrowheads, respectively. (D) *gli3* expression was not significantly affected in a stage-matched comparison.

The ZPA acts as an organizing center in the posterior limb/fin bud mesenchyme in part because it produces Shh [38], [40], [45], [50], [53]–[55]. Shh is required for limb A-P polarity, outgrowth and Fgf expression in the AER [55]. Zebrafish Shh (*shha*) and its receptor (and transcriptional target) *ptch2* are first expressed in the ZPA at 24 hpf and expression progressively increases until 36 hpf (Figure 3A–B) [56]. In Nipbl-deficient limb buds, *shha* and *ptch2* expression was reduced at these stages (Figure 3A, B). *shha* and *ptch2* expression levels were also reduced in the intestine (where Nipbl is also required for development [11]; Figure 3A, B, asterisks), but unaffected in the notochord and neural tube (Figure 3A, B and unpublished data), suggesting a tissue-specific requirement for Nipbl in the expression of Shh and its receptor.

Hand2 regulates *Shh* expression in fin/limb buds [56]–[58], and we found that *hand2* expression was also reduced in Nipbl-deficient fin buds (36 and 40 hpf) compared with stage-matched controls (32 and 36 hpf) (Figure 3C). In mouse limb buds, anterior expression of the transcriptional repressor, Gli3, restricts expression of *Hand2* posteriorly [59]. Zebrafish pectoral fin buds also express *gli3* [60] but its expression was not affected by reduction of Nipbl (Figure 3D).

**Figure 4:**
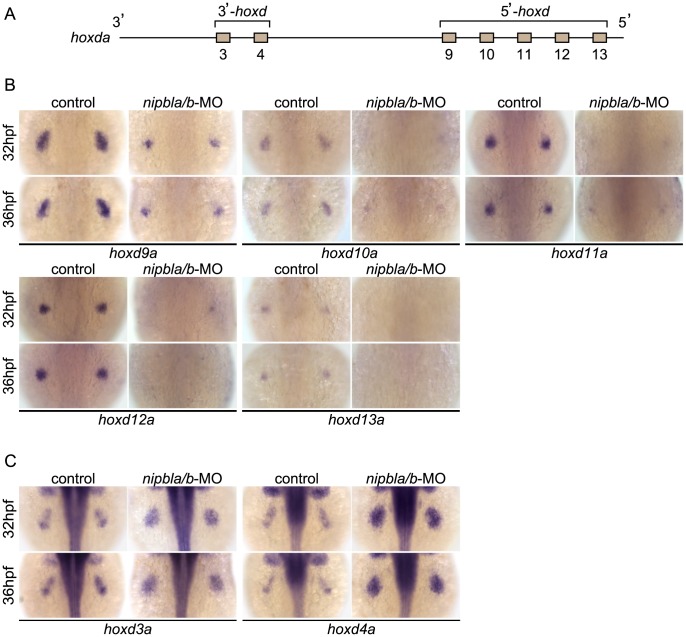
Nipbls are required for spatial patterns of *hoxd* expression in pectoral fin buds. (A) Diagram of zebrafish *hoxda* cluster. (B, C) Expression of 3′-*hoxd* genes including *hoxd3a* and *d4a* (B) and 5′-*hoxd* genes including *hoxd9a-d13a* (C) was examined by ISH at 32 and 36 hpf to show both time-matched and stage-matched (*nipbla/b*-MO at 36 hpf and control at 32 hpf) comparisons. Dorsal views, anterior to the top.

Mammalian Hand2 acts together with the products of 5′-*Hoxd* genes [58] in the regulation of *Shh* expression. In pectoral fin buds of Nipbl-deficient embryos, we found that 5′-*hoxd* genes, including *hoxd9a-d13a* (Figure 4A), were significantly downregulated (Figure 4B). Importantly, fin bud expression of *hand2*, *hoxd10a*, *shha* and *ptch2* could all be partially rescued by exogenous *nipbla* mRNA (Figure S8).

Retinoic acid (RA) produced in anterior somites also regulates *shha* expression in pectoral fin buds (12–22 hpf [42]–[44], [61]), as well as fin bud expression of *fgf10a*. However, we found no differences in expression of either the RA synthesizing enzyme *aldh1a2* or the RA degradation enzyme and target gene, *cyp26a1*, at 13 and 19 hpf in Nipbl-deficient embryos (Figure S9).

Together, these findings indicate that Nipbls regulate the *5′-hoxd/hand2/shha* signaling cascade, but do not affect the *tbx5a/fgf24/fgf10a* pathway that lies downstream of RA signaling, during vertebrate limb development.

### Nipbls regulate expression of *hox* genes according to their genomic location

*Hox* genes belong to 13 paralog groups organized in four (mammals) or seven (zebrafish) clusters; the *HoxA* and *D* clusters are crucial for limb/fin development [56], [62], [63]. The most 3′-located genes (3′-*Hox*), such as *Hoxd1*, are expressed earliest in mouse limb buds, whereas expression of 5′-located genes (*5′-Hox, d10-d13*) begins later [64], [65]. *5′-Hoxd* gene expression occurs first in proximal limb buds, where it is required for *Shh* expression in the ZPA to establish A-P patterning [55], [66], and is later restricted distally in limb buds, where it is required for proper digit formation [64], [65]. Expression of *hoxd* genes in zebrafish fin buds is reminiscent of that in proximal mouse limb buds but appears to lack the second wave of distal expression, consistent with the lack of digits in ray-finned fish [64].

**Figure 5:**
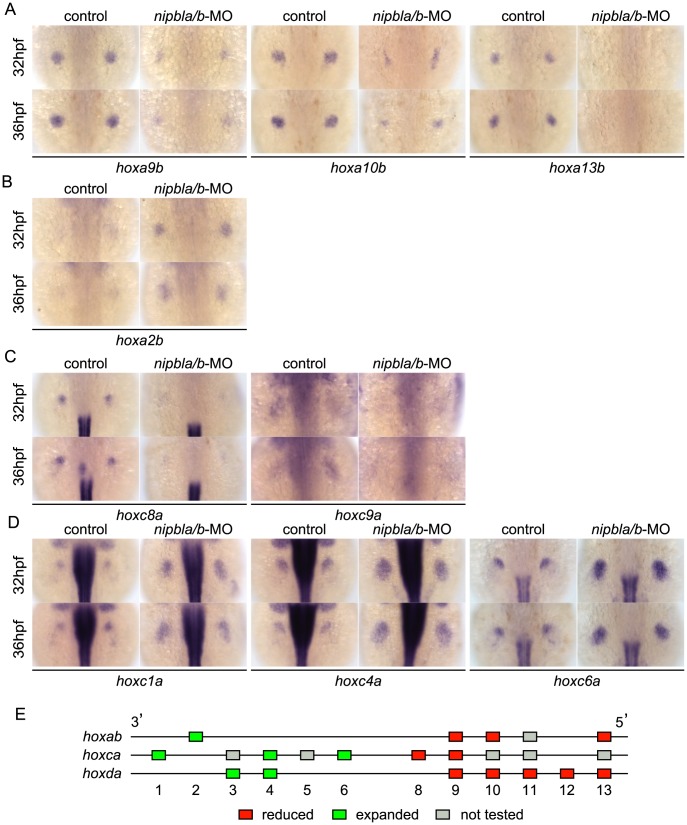
Nipbls regulate *hox* gene expression according to genomic location. (A–D) Expression of genes in *hoxab* (A,B) and *hoxca* (C,D) clusters was examined by ISH. (A) 5′-*hoxa*, (B) 3′-*hoxa*, (C) 5′-*hoxc*, and (D) 3′-*hoxc* genes. Dorsal views, anterior to the top. (E) Diagram summarizing effects of Nipbl reduction on *hox* genes. Genes located closer to 5′-ends show reduced expression (red boxes) whereas those closer to 3′-ends become expressed across entire fin buds (green boxes).

Examination of expression of multiple *hox* genes from the *Hoxa* (*hoxab*), *Hoxc* (*hoxca*), *and Hoxd* (*hoxda*) clusters in the fin buds of Nipbl-deficient embryos revealed that changes in expression correlated strongly with positions of genes within clusters (Figures 4–5). Expression of five *hoxd* genes located at the 5′ ends of the *hoxda* cluster (*hoxd9a-d13a*) was severely reduced (Figure 4B), while expression of two *hoxd* genes located more 3′ in the cluster, *hoxd3a* and *hoxd4a*, expanded to encompass the entire bud (Figure 4C). Similarly, expression of 5′-genes in the *hoxab* cluster—such as *hoxa9b*, *a10b*, and *a13b*—was significantly reduced in Nipbl-deficient fin buds, while a 3′ gene, *hoxa2b*, was upregulated (Figure 5A, B). Likewise, expression of *hoxc8a* and *hoxc9a* was reduced in Nipbl-deficient fin buds while expression of *hoxc1a*, *hoxc4a*, and *hoxc6a* expanded posteriorly (Figure 5C, D). Thus, in all three *hox* clusters expressed in the pectoral fin buds, expression of genes near the 3′ end of the cluster expands, whereas expression of those closer to the 5′ end is reduced (Figure 5E). Interestingly, this position-specific regulation of *hox* gene expression is specific to pectoral fin buds, since *hox* expression patterns in the neural tube were unaffected in Nipbl-deficient embryos (Figure S10).

Shh signaling from the ZPA regulates expression of several *hox* genes along the A-P axis of limb buds, and reduced expression of 5′-*hoxa*/*hoxd* genes as well as posterior expansion of *hoxc6a* expression, similar to that described above, occurs in Shh-deficient zebrafish [55]. To test if the Shh reductions resulting from Nipbl deficiency might cause the defects in *hox* gene expression, we treated wild-type embryos with the Shh signaling inhibitor, cyclopamine (CyA). Although CyA treatment caused some developmental delay, (∼4–5 hr, based on the A-P positions of pLL primordia [compare Fig. S11A with Fig. S5A], and no more than 12 hr based on pectoral fin development), it strongly reduced expression of *ptch2* as well as *hoxa13b*, *hoxd10a* and *hoxd13a*, while expression of *hoxc4a* and *hoxc6a* expanded posteriorly (compared with stage-matched controls, Figure S11B). These effects of CyA treatment resembled those of Nipbl depletion, but others did not - e.g. *hoxd4a* expression was severely reduced, and *hoxc8a* expression expanded posteriorly in CyA-treated embryos (Figure S11B), in contrast to Nipbl-deficient embryos (Fig. 4C, 5C). Thus, loss of Shh signaling cannot explain all of the changes in *hox* gene expression in Nipbl-deficient embryos, suggesting that either Nipbls regulate the expression of *hox* genes directly, or they do so via regulators other than (or in addition to) Shh.

### Gene expression changes in limb buds of *Nipbl*-haploinsufficient mice mirror those in Nipbl-deficient fish

**Figure 6:**
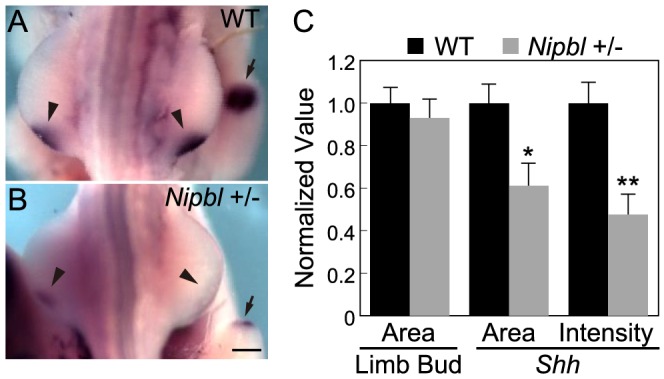
Reduced ZPA expression of *Shh* in *Nipbl* ^+/−^ mouse limb buds. (A–B) Whole mount ISH for *Shh* in the hindlimb buds of E10.5 wild type (A) and *Nipbl* ^+/−^ (B) mice. In these dorsal views, anterior to the top, the left and right ZPA are seen as localized patches of staining on the posteriolateral edge of each bud (arrowheads). The ZPA of the right forelimb bud is also visible in the background (arrows). Scale bar = 0.5 mm. (C) Quantification of ISH patterns from 5 wild type and 5 mutant embryos. Limb bud and ZPA size were estimated from image areas. Hybridization intensity was measured as mean pixel intensity in the ZPA multiplied by ZPA area. Data are normalized to wild type values. * = p<0.05, ** = p<0.01.

*Nipbl* ^+/−^ mutant mice fail to display obvious limb reductions, but do show some limb patterning and bone calcification defects [3]. Given the gene expression changes we found in pectoral fin buds of Nipbl-deficient fish, we decided to investigate if Nipbl-deficient mouse limb buds show some of the same changes. ISH for *Shh* in E10.5 limb buds of *Nipbl* ^+/−^ mice revealed a marked reduction in *Shh* expression in the ZPA, similar to Nipbl-deficient fin buds (compare Figure 3A and Figure 6). This was confirmed by both Q-RT-PCR and expression microarray analysis, using RNA extracted from E10.5 limb buds harvested from stage-matched *Nipbl^+/−^* (n = 12) and wildtype (n = 12) littermate embryos (Table 1; also see Methods). Microarray analysis identified approximately 1000 genes as significantly over- or under-expressed in *Nipbl* ^+/−^ limb buds (Table 1 and data publically deposited) and, similar to tissues and cells of *Nipbl* ^+/−^ mice and individuals with CdLS, most gene expression changes were typically less than 1.5-fold [3], [4]. Nonetheless, statistically-significant changes in expression (mostly decreases) were observed for multiple genes in the Fgf, Bmp and Shh pathways, as well as numerous genes in the Wnt/planar cell polarity signaling pathway. In addition, multiple genes at the 5′ and 3′ ends of the Protocadherin B cluster were downregulated (not shown), while *Stag1* (which encodes a cohesin subunit) was upregulated; both of these changes are hallmarks of Nipbl deficiency in other tissues [3].

**Table 1:** Gene expression changes in *Nipbl^+/−^* mouse limb buds.

	Expression by microarray		Expression by Q-RT-PCR			Expression by microarray		Expression by Q-RT-PCR	
Gene	FDR	mut/wt	mut/wt	Notes	Gene	FDR	mut/wt	mut/wt	Notes
**Shh pathway**									
*Shh*	1.0%	0.82	0.48 (p<0.01)		*Hmgb2*	8.6%	0.71		i
*Gli3*	9.5%	1.09			*Pbx2*	18.3%	1.03		ii
*Hhip*	2.4%	0.76			*Disp1*	1.0%	1.08		
*Hand2*	2.4%	0.92	0.85 (p = 0.09)	iii ^, ^ iv	*Ptch1*	1.0%	0.90	0.82 (p = 0.03)	
**FGF signaling**									
*Fgf15*	20.2%	0.91			*Ets1*	3.0%	0.93		v
*Fgf18*	1.8%	0.83		vi	*Ets2*	1.0%	0.92		
*Fgf9*	18.8%	0.88		vii	*Spred2*	1.0%	1.16		
*Spry2*	4.1%	0.89			*Gpc1*	2.4%	0.92		
**Hox Gene Expression and Function**									
*Hoxa13*	22.1%	0.73	0.46 (p = 0.03)		*Hoxd12*	1.0%	0.74	0.53 (p<0.01)	
*Hoxc9*	7.5%	0.89			*Hoxd13*	1.0%	0.65	0.51 (p<0.01)	
*Hoxc13*	2.4%	0.85	0.57 (p<0.01)		*Enpp2*	1.0%	0.75		viii
*Hoxd10*	1.0%	1.06	1.27 (p<0.01)		*Epha7*	1.0%	0.82		ix
*Hoxd11*	23.6%	0.93	0.75 (p = 0.04)		*Pbx1*	1.0%	1.12		x
**BMP signaling**									
*Bmp2*	1.0%	0.79		iv	*Bambi*	1.0%	0.89		
*Bmp4*	1.0%	0.90		iv	*Rgmb*	3.5%	0.90		
*Bmp7*	6.4%	1.10			*Msx2*	9.9%	1.10		
*Bmpr1a*	1.0%	1.10							
**Cohesin Function**									
*Nipbl*	1.0%	0.63	0.62 (p<0.01)		*Esco1*	22.3%	1.13		
*Stag1*	1.0%	1.16							
**Mediator Complex**									
*Med12l*	1.8%	1.11			*Med19*	1.0%	1.09		
*Med14*	4.1%	1.05							
**Wnt/Planar Cell Polarity Pathway**									
*Wnt11*	1.0%	0.87		xi	*Csnk2a1*	1.0%	1.09		
*Wnt8a*	9.9%	1.12		xii	*Csnk2a2*	7.5%	1.05		
*Fzd2*	2.4%	0.92			*Cthrc1*	1.0%	0.72		xiii
*Fzd8*	1.0%	0.86			*Gpc4*	1.0%	1.14		
*Dkk2*	1.0%	0.82			*Gpc6*	1.0%	1.16		
*Rspo2*	1.8%	0.84		xiv	*Daam1*	8.6%	0.91		
*Rspo3*	1.0%	0.74			*Daam2*	1.0%	0.75		
*Sfrp1*	4.6%	0.81		iv	*Nlk*	1.0%	1.15		
*Sulf1*	1.0%	0.81			*Ppap2b*	1.0%	0.85		
*Fat1*	1.0%	0.89			*Prickle1*	1.0%	0.91		
*Fat3*	1.0%	0.78			*Ror1*	2.4%	0.88		
*Fat4*	1.0%	0.65							

Relative gene expression levels in limb buds of stage-matched, E10.5 wildtype and *Nipbl^+/−^* mice were determined as described (see Materials and Methods), and in certain cases confirmed by Q-RT-PCR. Selected transcripts are shown.

i Involved in control of *Shh* expression in limb bud [101].

ii Also controls *Hox* gene expression in the limb bud [102].

iii Also controls *Hox* gene expression in the limb bud [69].

iv Direct target of Hoxd13 in limb buds [103].

v Directs the position of the *Shh* expression boundary delineating the experimentally defined ZPA [104].

vi Mesenchymal, involved in chondrocyte proliferation [105].

vii AER-*Fgf* [106].

viii Strongly activated by HOXA13 [67].

ix HOXA13 target in limb buds [68].

x Functions as a HOX cofactor during development; complexes with HOXA9; also controls *Hox* and *Shh* expression [102].

xi Non-canonical Wnt [107].

xii Canonical Wnt [108].

xiii Interacts with some Wnts and Frizzleds and supports Wnt-Fz-Ror2 complex formation, and at the same reduces Wnt-Fz-LRP complex formation, thus favoring non-canonical Wnt signaling [109].

xiv Wnt regulator; required for maintenance of AER and Shh signaling [110].

Similar to Nipbl-deficient fin buds, *Nipbl^+/−^* limb buds displayed reductions in the expression of 5′-*Hox* genes (Table 1 and Figure S12). This was particularly obvious for genes at the extreme 5′ end of *Hox* clusters, such as *Hoxa13*, *Hoxc13*, *Hoxd12*, and *Hoxd13*, expression of which was reduced between 15% and 35% by microarray, although Q-RT-PCR measurements (Table 1) and ISH (Figure S12) suggested that the true decrease is probably closer to 50%. Also downregulated were *Enpp2* and *Epha7*, which are known targets of 5′-*Hox* genes (Table 1 and [67], [68]). *Hand2*, which lies upstream of both *Shh* and *Hox* gene expression [58], [69], was also modestly downregulated (Table 1) similar to Nipbl-deficient fish fin buds (Figure 3C).

Overall, reductions in 5′-*Hox* gene expression in Nipbl-deficient mouse limb buds were not as large as those observed in Nipbl-deficient zebrafish, most likely reflecting the fact that Nipbl expression is more severely reduced in MO-injected fish embryos than in haploinsufficient mice (which, due to compensatory mechanisms, only show a 37% reduction in *Nipbl* transcript levels; cf. Table 1). Nonetheless, at least for the *HoxD* cluster, the downregulation of the most 5′- genes in *Nipbl^+/−^* mouse limbs was accompanied by upregulation of at least some genes lying more 3′ in the same cluster (Figure S12A–D).

### Med12 and Nipbl regulate spatial expression of *hox* gene expression and act together in pectoral fin development

**Figure 7:**
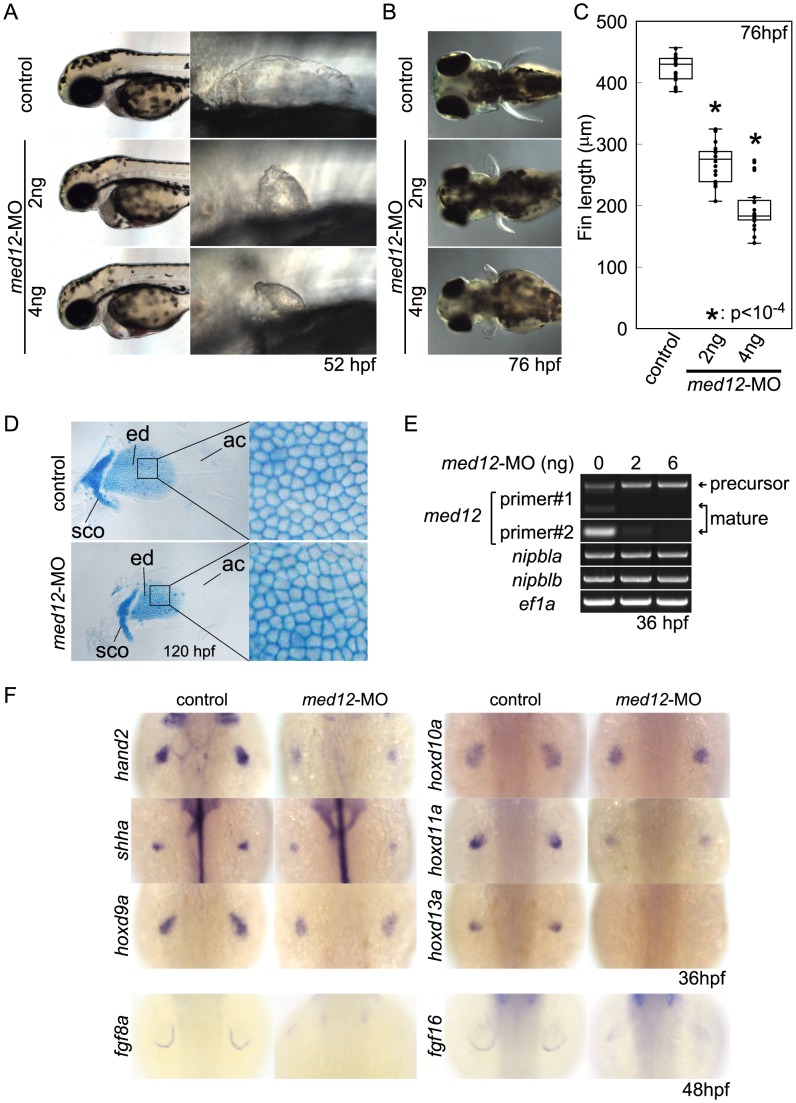
Med12 depletion disrupts pectoral fin morphology and gene expression similar to Nipbl depletion. (A, B) Morphology of live embryos at 52 hpf (A, lateral views) and 76 hpf (B, dorsal views). (A) Anterior halves of control and *med12*-MO-injected embryos (left column) and higher magnification pictures of their pectoral fin buds (right column). (B) Dorsal views of embryos at 76 hpf. (C) Whisker plots of fin length. Fin lengths (medians) are 430.0 µm, n = 18 (control), 275.6 µm, n = 20 (*med12*-MO, 2 ng), and 183.8 µm, n = 20 (*med12*-MO, 4 ng). *: p<10^−4^. (D) Alcian blue staining of pectoral fin cartilage of control (upper) and Med12-deficient (*med12*-MO, 4 ng; lower) embryos at 120 hpf. Dorsal view, anterior to the top. Right column, higher magnification pictures of boxed areas of endoskeletal discs. ac, actinotrichs; ed, endoskeletal disc; sco, scapulocoracoid. (E) Controls for *med12*-MO efficiency. RT-PCR, 36 hpf. Both pairs of *med12* primers (Primer #1 and #2) show that splicing of *med12* mRNA is significantly suppressed by *med12*-MO, with a slightly higher efficiency at 6 ng. Primer pair #1 detects both precursor and mature mRNA, whereas primer pair #2 only detects mature mRNA (see Materials and Methods). *nipbla* and *nipblb* expression was not affected by Med12 depletion. *ef1a* was used as a control. (F) Expression of genes involved in the 5′-*hox*/*hand2*/*shha* gene cassette and AER *fgf* genes in pectoral fin buds examined by ISH at 36 hpf. Dorsal views, anterior to the top. Similar to Nipbl-deficient embryos, *shha*, *hand2* and 5′-*hoxd* genes in mesenchyme as well as *fgf16* and *fgf8a* in the AER are reduced in Med12-deficient embryos (4 ng/embryo *med12*-MO).

**Figure 8:**
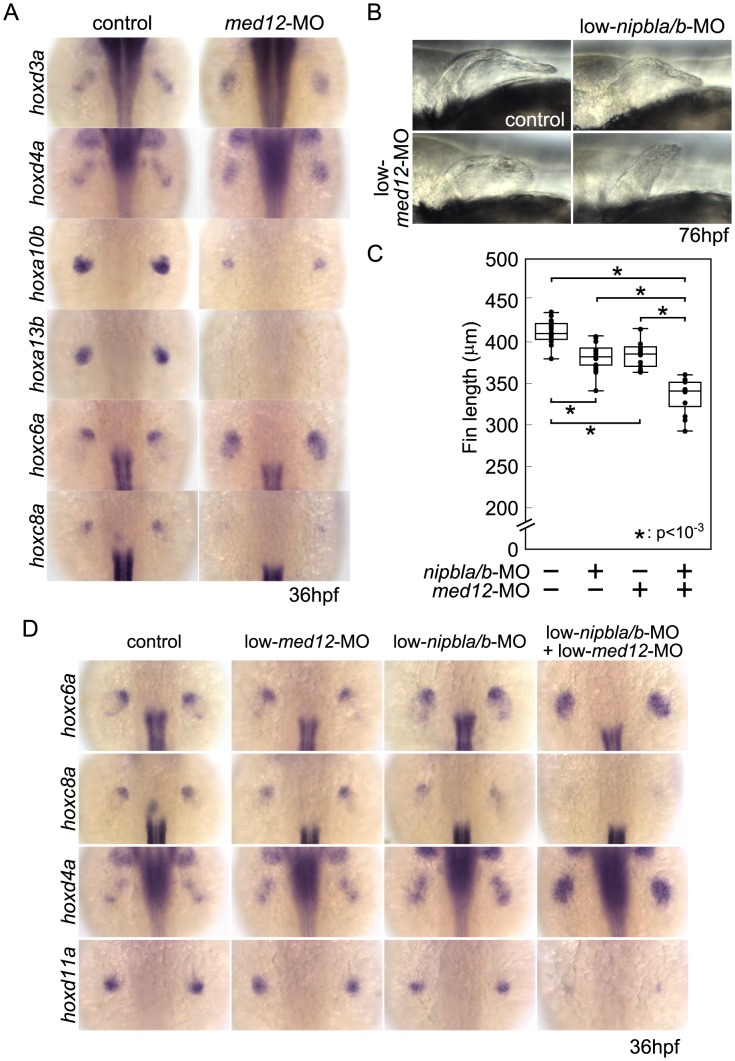
Functional interactions between Nipbl and Med12 in pectoral fin development. (A) *hox* gene expression in pectoral fin buds of Med12-deficient embryos examined by ISH at 36 hpf. Dorsal views with anterior to the top. (B) Lateral views of pectoral fins in living larvae at 76 hpf in controls or injected with 0.5 ng *med12*-MO alone (low-*med12*-MO). (C) Pectoral fin lengths in larvae injected with low-*med12*-MO alone or combined with low amounts of *nipbl*-MOs (0.05 ng *nipbla*-MO+0.75 ng of *nipblb*-MO; low-*nipbla/b*-MO). Medians: 410.1 µm, n = 16 (control), 382.2 µm, n = 24 (low-*nipbla/b*-MOs), 385.4 µm, n = 16 (low-*med12*-MO alone), and 341.4 µm, n = 16 (low-*med12*-MO+low-*nipbla/b*-MOs). Asterisks indicate statistical significance (p-values<0.001). (D) *hox* expression in larvae injected with low-*med12*-MO alone or combined with low *nipbla/b*-MO. Dorsal views, anterior to the top.

Recent studies indicate that Nipbl and cohesin can co-localize at enhancer and promoter regions with the Mediator complex, suggesting that Nipbl participates with Mediator in linking distant transcriptional regulators to basal transcriptional machinery [17]. Interestingly, in zebrafish, a loss-of-function mutation in *med12*, which encodes a subunit of Mediator, disrupts pectoral fin development [37]. We injected embryos with varying amounts of *med12*-MO ([70]; up to 6 ng/embryo), and observed severe reductions in pectoral fins at 52–120 hpf (Figure 7A–E) that resembled Nipbl-deficient embryos. Moreover, Med12 depletion caused changes in gene expression in pectoral fin buds strikingly similar to those observed in Nipbl-deficient embryos (Figures 7F and S13), particularly changes in expression of *hox* genes. Notably, the same 3′ genes of the *hoxab*, *hoxca* and *hoxda* clusters were expanded posteriorly following knockdown of *med12*, while expression of the same 5′ genes was reduced (Figure 8A).

The possibility that these similarities reflect a transcriptional relationship between Nipbl and Med12—e.g. Nipbl positively regulates Med12 expression (or vice versa)—was ruled out by direct measurements of transcript levels in the fin buds of MO-injected embryos (Figures 7E and S14). This conclusion also agrees with the mouse microarray data, which show no decrease in expression of any Mediator subunit in *Nipbl* ^+/−^ limb buds. Indeed, some Mediator genes (*Med14*, *Med19*, and *Med12l*) exhibit modest increases in expression, suggesting, if anything, a negative role for Nipbl in Mediator expression (Table 1).

To test for a genetic interaction between Nipbl and Mediator, *nipbla/b*-MOs and *med12*-MO were co-injected at subthreshold doses, and assayed for changes in pectoral fin development and gene expression. Small amounts of *med12*-MO (0.5 ng/embryo; low-*med12*-MO) caused only slight reductions in pectoral fin size and 5′-*hoxa*/*hoxd* gene expression in fin buds (Figure 8B–D). However, when combined with low doses of *nipbla/b*-MOs (a combination of 0.05 ng/embryo of *nipbla*-MO and 0.75 ng/embryo of *nipblb*-MO; low-*nipbla/b*-MO), low-*med12*-MO caused reductions in 5′-*hox* gene expression and expansion of 3′-*hox* gene expression similar to those observed with higher doses of either *nipbla/b*- or *med12*-MOs alone (Figure 8D). These results suggest that Nipbl and Mediator interact functionally to regulate spatial patterning of *hox* gene expression in the developing limb.

Interestingly, depletion of the cohesin subunit Rad21 caused very different defects in pectoral fin development and gene expression than deficiencies for Nipbl or Med12. Rad21 depletion delayed development (by approximately 10 hrs, based on the A-P positions of pLL primordia; Figure S15), consistent with a previous report [71], but when compared with stage-matched controls all fin mesenchymal genes (including 3′-*hox* genes, *hoxc6a* and *hoxd4a*) were downregulated (Figure S16). Reductions in *hox* gene expression became more severe at later stages, although, interestingly, only in fin buds, and not in the neural tube (Figure S16).

### Nipbl and Med12 regulate chromatin conformation around the *hoxda* cluster

**Figure 9:**
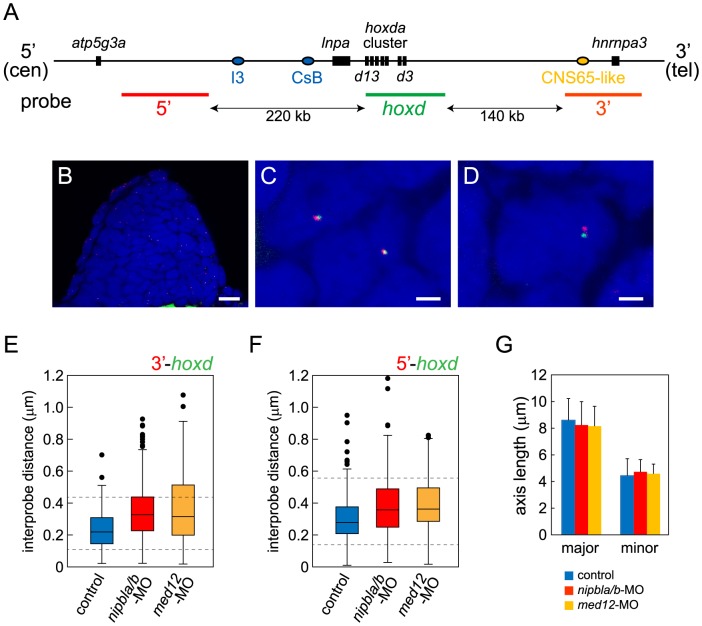
Nipbls and Med12 play roles in regulation of higher-order chromosome conformation at the Hoxd locus in pectoral fin buds. (A) Diagram of the genomic organization at the zebrafish *hoxda* locus. Genes in the *hoxda* cluster and flanking genes are shown as black boxes. Putative regulatory elements conserved between zebrafish and mouse and probes used for FISH are shown as colored ovals and lines, respectively. (B–D) Typical images of FISH. (B) Low magnification picture of a sagittal section of pectoral fin bud. Scale bar = 10 µm. (C,D) Higher magnification images of nuclei with colocalized (C) and separate signals (D). Hybridized probes are detected as green and red fluorescent dots in DAPI-stained nucleus. Scale bar = 2 µm. (E,F) Whisker plots of interprobe distances between *hoxd* and 3′ probes (E) or *hoxd* and 5′ probes (F) at 38 hpf. Medians, numbers of nuclei and embryos, and p-values calculated by the non-parametric Mann-Whitney U-test are shown in Table 2. Dotted lines indicate thresholds for separated (upper) and closed (lower) signals in Table 2. (G) Sizes of nuclei in pectoral fin buds (n = 30 each) were estimated at 38 hpf by measuring major and minor axes. Major axis (Ave ± S.D.): 8.58±1.63 µm (control), 8.22±1.76 µm (*nipbla/b*-MOs, p = 0.412), and 8.14±1.43 µm (*med12*-MO, p = 0.280). Minor axis (Ave ± S.D.): 4.41±1.28 µm (control), 4.70±0.92 µm (*nipbla/b*-MOs, p = 0.314), and 4.56±0.73 µm (*med12*-MO, p = 0.577). p-values were calculated by Student's t-test.

Spatial- and temporal patterns of *Hox* gene expression are achieved through regulation of chromatin organization around *Hox* clusters. In mouse limb buds, for example, remote enhancers located in flanking “gene deserts” found at the telomeric (3′) and centromeric (5′) sides of the clusters regulate the proximal versus distal expression of *5′-Hox* genes [15], [72] ; these enhancers are distinct from cis-regulatory elements within the clusters that regulate co-linear expression along the body axis [73], [74]. Although these remote enhancers have been most extensively studied in mammals, some are clearly conserved and functional in teleosts [75]–[77]. For example, of two distinct regions in the gene desert telomeric to the mouse *HoxD* cluster recently shown to have proximal limb-specific enhancer activity [72], we located sequences homologous to one, CNS65, about 200 kb telomeric to the *hoxda* cluster in the zebrafish genome (Figure 9A).

Such results suggest that, in both fish and mice, limb bud *hox* gene expression depends on long-range chromosomal interactions the formation of which may be regulated by Nipbl and Mediator [17]. We tested this hypothesis by looking for changes in chromatin architecture around the *hoxda* cluster following Nipbl or Mediator depletion, using probes for 3D-FISH with which we can measure physical distances between the *hoxda* cluster and distant flanking regions on both centromeric and telomeric sides (Figure 9). When 3D-FISH was performed on cryosections of 38 hpf pectoral fin buds, using a *hoxd* probe and a distant flanking probe (either 3′ or 5′), each nucleus typically contained one or two pairs of closely-spaced fluorescent spots (Figure 9B–D). The separation between spots varied among nuclei (Figure 9E, F), even within a single fin bud, consistent with the dynamic nature of chromatin interactions [78], [79]. However, when measured in large numbers (≥180 nuclei per condition) we observed a significant increase in inter-probe distances in both Nipbl-deficient and Med12-deficient fin buds, both when centromeric and telomeric flanking probes were used (Figure 9E, F, Table 2). Indeed, a significant percentage of Nipbl- and Med12-deficient nuclei showed inter-probe distances more than double those of controls, and the number of nuclei with probes in close proximity was significantly reduced (Table 2). These effects were not due to changes in nuclear size (Figure 9G). Overall, the results strongly suggest that Nipbl and Mediator regulate expression of *hoxd* genes in developing limbs by modulating the interaction of promoters with remote enhancers.

**Table 2:** Results from 3D-FISH around the zebrafish *hoxda* locus.

	median (µm)	nuclei (embryos)	p*	% of nuclei**	
				closed	separated
5′-*hoxd* (centromeric)					
pectoral fin buds					
control	0.278	240 (4)		9.58	5.00
*nipbla/b*-MO	0.357	240 (4)	4.8×10^−7^	6.25	15.9
*med12*-MO	0.362	180 (3)	7.9×10^−9^	5.56	17.2
hindbrain					
control	0.295	165 (4)		8.48	3.64
*nipbla/b*-MO	0.312	160 (4)	0.092	8.12	8.12
*med12*-MO	0.306	125 (3)	0.716	9.60	2.40
3′-*hoxd* (telomeric)					
pectoral fin buds					
control	0.220	240 (4)		9.58	1.67
*nipbla/b*-MO	0.328	420 (7)	5.5×10^−19^	4.76	25.0
*med12*-MO	0.317	180 (3)	2.2×10^−9^	6.11	28.3
hindbrain					
control	0.237	160 (4)		15.0	6.25
*nipbla/b*-MO	0.349	160 (4)	2.2×10^−11^	4.38	26.9
*med12*-MO	0.337	160 (4)	1.3×10^−8^	3.13	21.9

* Evaluated by the Mann-Whitney test.

** Proportions of nuclei exhibiting interprobe distances less than half of (closed) and longer than double (separated) the control medians.

We also used 3D-FISH to examine chromosome conformation at the *hoxda* cluster in cells of the hindbrain, where Nipbl deficiency does not alter *hox* gene expression (Fig. S10). Similar to pectoral fin buds, we observed close apposition between the *hox* cluster and its 3′- and 5′- flanking regions (Figure S17, Table 2), which agrees with data showing that long-range *HoxD* interactions in the mouse occur in both the limbs and the CNS [15], [77], [80]. Interestingly, while Nipbl- or Med12-depletion both increased the separation between the *hox* cluster and 3′ flanking sequences in the CNS (similar to the fin buds), they did not alter separation between the *hox* cluster and 5′ flanking sequences in the hindbrain. The potential significance of these results is discussed below.

## Discussion

### Multiple genes are dysregulated in fin/limb buds of Nipbl-deficient embryos

Limb reductions are among the most striking structural birth defects in CdLS [26], [28], [36]. Previous studies of both fish and mouse models of Nipbl deficiency, as well as of cell lines derived from human patients with CdLS, strongly suggest that such defects result from the collective and sometimes synergistic effects of numerous small changes in gene expression during development [3], [4], [11]. Distinct sets of gene expression changes have been found in every tissue studied thus far, providing insights into genetic pathways that underlie defects in different tissues and organs [3], [4], [11]. Until now, identifying gene expression changes underlying limb reductions in CdLS has not been possible, since limb reduction is one of the few structural defects in CdLS that is not obviously replicated in the *Nipbl*-haploinsufficient mouse model [3]. However, by combining studies of zebrafish and mice in the present study, we show that Nipbl levels are critical for limb development (Figure 1), and that Nipbl regulates expression of specific sets of genes in the embryonic limb, including many key developmental regulators that are conserved between fish and mice. Among these *Fgfs*, *Shh*, and 5′-*Hox* genes (Figures 2, 3, 5, and Table 1) are of particular note because of the central and conserved roles these genes play in early limb bud growth and patterning.

In the E10.5 mouse embryo, where the larger size of the limb bud (compared with zebrafish) made genome-wide transcriptional profiling feasible, levels of more than 1000 transcripts were significantly altered (Table 1 and data publically deposited). Both the large number of affected genes and the relatively small sizes of the effects were similar to what has been observed in other tissues of *Nipbl* ^+/−^ mice and in cells from individuals with CdLS [3], [4]. It may be noteworthy that in the mouse limb a large number of Nipbl-sensitive genes are involved in Wnt/planar cell polarity signaling. Although this finding was not further investigated here, it is possible that disruption of this pathway is related to the disorderly arrangement of endoskeletal cells that we consistently observe in developing, Nipbl-deficient fins (Figure 1F′, G′). It may also be noteworthy that, in Nipbl-deficient mouse limbs, several Mediator subunits are (slightly) upregulated (Table 1). As described below, upregulated Mediator function might potentially provide some compensation for Nipbl deficiency.

### Interactions between Nipbl and Mediator in gene regulation

Chromatin binding studies have shown that Nipbl co-localizes with cohesin and the Mediator complex at putative regulatory elements of actively transcribed genes, suggesting that Nipbl and Mediator act together to regulate gene expression [13], [17], [81]. Here we provide the first in vivo evidence in support of this hypothesis: 1) Med12- and Nipbl-deficient pectoral fin buds display similar size reductions and gene expression changes—particularly within *hox* gene clusters; 2) subthreshold doses of *nipbl*- and *med12*-MOs synergize to reduce limb size and disrupt gene expression; and 3) both *nipbl*- and *med12*-MOs cause similar changes in chromatin conformation at the *hoxda* locus.

These results support the view that Nipbl and Mediator play roles in the long-range coordination of gene expression. Moreover, the observed differential effects on expression of 3′- versus 5′-*hox* genes suggest an important role for Nipbl and mediator in transcriptional coordination at multi-gene loci, a result also supported by position-specific effects seen at the protocadherin beta locus in *Nipbl*-haploinsufficient mice [3], and by studies on the role of Nipbl in long-range control of the beta-globin locus [13].

Interestingly, instead of having position-specific effects, depletion of the cohesin subunit Rad21 led to downregulation of all 3′- and 5′-*hox* genes that we tested, suggesting that the gene regulatory effects of Nipbl/Mediator are not equivalent to those of cohesin. Indeed, although cohesin has been implicated in long-range chromatin interactions [82]–[84], and Rad21 co-localizes at promoters and enhancers with Nipbl and Mediator [17], this co-localization only occurs at a subset of cohesin binding sites. Moreover, recent work suggests that Nipbl, but not cohesin, co-localizes with certain transcription factors [85]. Such differences may explain the markedly different results that have been observed, in both cell lines and embryos, in the changes in gene expression and chromatin organization that occur in response to depletion of cohesin versus Nipbl [11], [85], [86].

### Direct versus indirect effects of Nipbl and Mediator in limb development

Previous studies have proposed that limb development is controlled by a positive feedback loop in which Shh from the ZPA and Fgfs from the AER maintain one another's expression [38], [40], [53]. Consistent with this, we found that expression of both *Shh* and *Fgf* genes were reduced in Nipbl-deficient limb and fin buds (Figures 2, 3 and Table 1). As *nipbla* and *nipblb* are expressed most highly in fin bud mesenchyme (Figure S1), it is possible that Nipbls regulate the expression of mesenchymal genes such as *shha* directly, whereas regulation of *fgf* expression in the AER may be indirect.

On the other hand, *hox* genes could be the major direct targets of Nipbl deficiency, with effects on *shha* expression being secondary. Both HoxD and Hand2 regulate *Shh* expression in early limb/fin buds [57], [58], [87], and Hox proteins also regulate *Hand2* expression [88]. In *Drosophila*, Nipped-B and cohesin bind to genes in the *bithorax* (*Hox*) complex (BX-C), specifically in cells that express BX-C genes [81]. More recently, it has been shown that human cohesin binds to the *HOXA* and *HOXB* clusters, and disruption of its function reduces expression of multiple *HOX* genes [83]. Our finding that three distinct *hox* clusters (A, C, and D) are all affected similarly in Nipbl- and Med12-deficient zebrafish suggests that Nipbl and Mediator play a common role in *hox* locus control. Results of 3D-FISH experiments at the *hoxda* cluster further suggest that Nipbl/Mediator-dependent regulation of long-range chromatin interactions is an important part of this role, as discussed below.

### Regulation of chromatin conformation by Nipbl and Mediator

**Figure 10:**
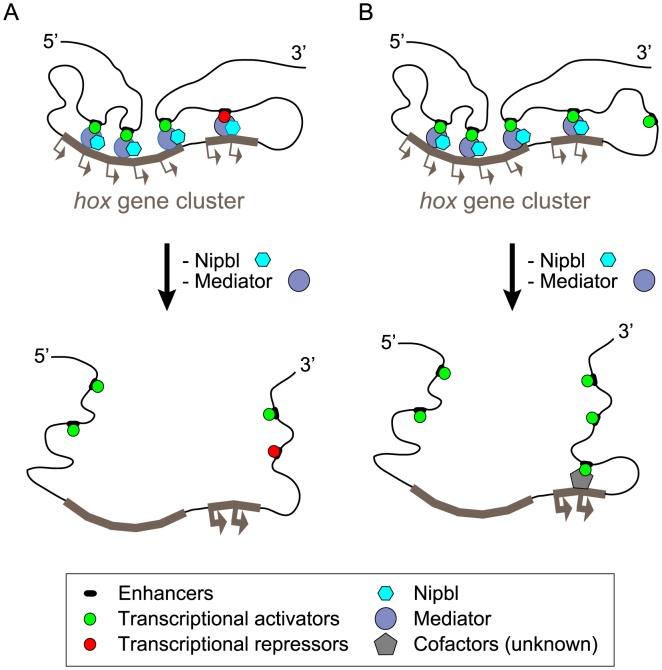
Model of *hox* gene regulation by Nipbls and mediator. Along topological domains, 3′- and 5′-*hox* genes tend to interact with limb-specific regulatory elements in telomeric and centromeric landscapes, respectively. These interactions are required to establish proper patterns of *hox* gene expression in limb/fin buds and depend on Nipbl/Mediator. The long-range enhancer-promoter interactions are disrupted in the absence of Nipbl and Med12, leading to dysregulation of *hox* genes. (A) Expanded expression of 3′ -*hox* genes might be allowed when released from putative remote repressors in Nipbl/Med12-deficient fin buds. (B) Alternatively, disruption of chromosomal conformation may lead to replacement of 3′ remote enhancers with (more closely located) putative ectopic enhancers that can activate 3′-*hox* genes strongly through long-range interactions.

The position-specific effects of depleting Nipbl or Med12 on *hox* gene expression in the zebrafish pectoral fin bud—with 5′-genes down-regulated and 3′ genes up-regulated—suggest a coupling of transcriptional regulation between the two ends of *hox* clusters. Our 3D-FISH results, which show that Nipbl and Med12 are required in fin buds for long-range interactions on both sides of the *hoxda* cluster, raise two possibilities for explaining the effects of depleting Nipbl and Med12 on *hox* gene transcription (Figure 10).

According to one model, disruption of long-range chromosomal interactions leads to a loss of long-range activation at the 5′ ends and long-range repression at the 3′ ends of *hox* clusters (Figure 10A). Alternatively, disruption of chromosomal conformation may allow the 3′ remote enhancers to be replaced with other (probably more closely located) regulatory elements, leading to their ectopic activation (Figure 10B). These putative regulatory elements might be fish-specific since, the orthologous 3′-*Hox* genes are not upregulated in Nipbl-deficient mouse limb buds.

On the other hand, direct comparisons between mice and fish could be misleading, due to the dynamics of *hox* gene expression. In both tetrapod and zebrafish limb buds, *hox* gene expression progresses through distinct stages, first being biased toward 3′ genes and later toward 5′ ones [64], as the balance of long-range interactions shifts from telomeric to centromeric [72], [77]. If E10.5 mouse hindlimb buds are not at exactly the same stage as the pectoral fin (forelimb) buds examined here, they may not possess the same potential to express 3′-*Hox* genes.

A third possibility is that some upregulation of *Hox* genes does take place in the *Nipbl*-deficient mouse limb, similar to fish, but the genes affected are not as close to the 3′-end of the cluster. For example, among *Hoxd* genes, we observed that significant up-regulation of *Hoxd10*, and possibly also *Hoxd8*, accompanies the down-regulation *Hoxd11*, *12* and *13* in *Nipbl* ^+/−^ limbs.

Interestingly, in the zebrafish hindbrain, the effects of depletion of Nipbl or Med12 on *hox* gene expression and chromosomal interactions differ from those observed in fin buds. 3D-FISH in wild-type hindbrain cells reveals chromosomal interactions of *hoxda* with both 3′- and 5′-territories—despite the fact that the hindbrain expresses only 3′-*hox* genes. Moreover, the expression of hindbrain *hox* genes is unaffected in Nipbl- or Med12-deficient embryos, even though long-range interactions on the 3′ side of the *hoxda* cluster are markedly diminished. These results suggest: 1) that hindbrain *hox* gene expression is not primarily controlled by long-range enhancers (at least not on the 3′ side), and 2) that long-range interactions of *hox* genes are not necessarily associated with active transcription (i.e. they may sometimes represent a poised, or latent state). Consistent with the latter idea, in mouse forebrain, where *Hox* genes are not expressed, the *Hoxd* locus still interacts with many of the same long-range elements as it does in limb bud cells [15]. Similar examples of long-range promoter-enhancer associations that do not necessarily correlate with gene expression have also been described for *Shh* in the mouse limb [14].

Whereas Nipbl and Med12 depletion inhibits both 3′- and 5′- chromosomal interactions of the *hoxda* cluster in the pectoral fin buds, in the hindbrain such depletion fails to affect 5′ interactions, suggesting a distinct underlying mechanism. In the trunk, the activation of *hox* gene expression is thought to reflect an anterior-to-posterior wave of chromatin decompaction, from 3′ to 5′, such that in anterior structures (such as the hindbrain) 5′-*hox* genes and adjacent sequences remain in a highly condensed state [89], associated with high levels of H3K27me3 modification [80]. One possible interpretation of our results is that Nipbl and Med12 play essential roles in long-range interactions, but are not required for the maintenance of the condensed state. The full explanation, however, is likely not this simple, in view of a recent study involving siRNA-treated cell lines and cells from CdLS patients, which shows that chromatin compaction at some loci is highly sensitive to reductions in Nipbl function [86]. Interestingly, the effects on compaction reported in that study were not reproduced by knockdown of the Smc3 cohesin subunit, underscoring the idea, discussed earlier, that the transcriptional function of Nipbl is distinct from that of cohesin.

### Understanding the variability of limb defects caused by Nipbl deficiency

Many of the mesenchymal genes (e.g. *shh*, *hand2*, 5′-*hox*) we find downregulated in Nipbl-deficient fin buds are essential for growth and patterning of mouse limbs. *Shh* ^−/−^ mice, for example, have limb truncations [90], [91], and *Hand2* is required for *Shh* expression in the ZPA [87]. Mice lacking certain genes within the *HoxA* or *HoxD* clusters have mild digit defects, while a simultaneous deletion of both *HoxA* and *HoxD* clusters causes dramatic forelimb truncations [62], [63]. Our finding that expression of *Shh* and multiple *Hox* genes is reduced in the limb buds of *Nipbl* ^+/−^ mutant mice indicates that these genes are common targets of Nipbl in the vertebrate limb, and the dysregulation of their expression is likely to be central to the etiology of limb defects in CdLS.

Nonetheless, *Nipbl*+/− mice display very mild limb abnormalities [3]. One likely explanation for this difference is that haploinsufficiency does not lower Nipbl levels as much as is achieved in MO-injected zebrafish embryos. Indeed, it has been observed that, due to unknown compensatory mechanisms, *Nipbl*+/− mice display only a 35–40% reduction in *Nipbl* transcripts (cf. [3], and Table 1), whereas *nipbl* MOs can lower *nipbla* and *nipblb* transcript levels to a much larger degree [11].

The idea that the strength of limb phenotypes is related to the degree of *nipbl* depletion is further supported by the observation, in zebrafish, that fin reductions are more severe when larger amounts of *nipbla*-MO are injected, or when both *nipbla* and *nipblb* are knocked-down, as opposed to either one alone (Figure S3). In light of this observation, it is noteworthy that only about a third of individuals with CdLS display limb abnormalities at the severe end of the spectrum [26]. A subset of this phenotypic variability likely relates to the strengths of different mutations on Nipbl protein expression (severe forelimb defects tend to correlate with nonsense or frame shift mutations [92], [93]). However, it likely also reflects inter-individual variability in the functions of genes that control Nipbl expression or, like components of the Mediator complex, work together with Nipbl in the control of gene expression.

## Materials and Methods

### Ethics statement

All animals were handled in strict accordance with good animal practice as defined by the relevant national and/or local animal welfare bodies, and all animal work was approved by the University of California, Irvine, Institutional Animal Care and Use Committee.

### Fish and mouse maintenance, embryo raising and staging

Zebrafish (AB strain) were maintained and staged as described [94], [95]. Embryos were stage-matched based on relative positions of posterior lateral line primordial along the A-P axis, detected by ISH with a *fgf10a* probe. Pectoral fin buds and the posterior end of the yolk sac extension were used as landmarks (Figure S5). *Nipbl^+/−^* (RRS strain) mice were housed, mated, and staged as described previously [3].

### Microinjection of morpholino antisense oligonucleotides (MOs) and mRNA

MOs were designed to block translation (Gene Tools, Inc.), prepared at 20 mg/ml and diluted in 1× Danieau buffer [58 mM NaCl, 0.7 mM KCl, 0.4 mM MgSO_4_, 0.6 mM Ca (NCO_3_)_2_, 5 mM HEPES (pH 7.6)] and stored at −20°C. MO sequences are shown elsewhere (all *nipbl*-MOs and *rad21*-MO [11], and *med12*-MO [70]).

Full-length cDNA of *nipbla* was prepared by fusing partial cDNA fragments amplified by RT-PCR in pCRII-TOPO, fused with SV40 polyA sequence derived from pCS2+ and subcloned into pBS-KS+ for in vitro mRNA synthesis. Full-length capped *nipbla* mRNA was synthesized using mMESSAGE mMACHINE (T3) kit (Ambion) in the presence of rGTP according to the manufacturer's instructions. Synthesized mRNA was electrophoretically separated and a full-length mRNA was gel-isolated using RECOCHIP (TAKARA). MOs and full-length *nipbla* mRNA were injected into embryos at the 1–4-cell stage. A combination of *nipbla*-MO and *nipblb*-MO were injected to generate Nipbl-deficient embryos, at 0.75 ng/embryo each or otherwise as indicated in figure legends.

### Whole mount in situ hybridization (ISH)

Whole mount ISH of zebrafish embryos was performed using digoxigenin (DIG)-labeled antisense RNA probes as previously indicated [11]. Whole mount ISH of E10.5 mouse embryos was performed according to published protocols [96]. The 642 bp mouse Shh probe has been previously described [97]. The *Hoxd12* and *Hoxd13* probes were a kind gift from Denis Duboule.

### Measurement of fin length

Pectoral fin lengths were measured using ImageJ from the proximal base to the distal tip in dorsal views (Whisker plots). The interquartile ranges (IQR) are shown as boxes, with the median as the horizontal lines within the boxes. The upper and lower whiskers are the highest and lowest data points within 1.5× the IQR from the top and bottom of the box, respectively. Individual data including outliers are shown as dots. p-values are calculated by the non-parametric Mann-Whitney U test with the Bonferroni adjustment.

### RNA preparation and RT-PCR

Total RNA was extracted from 20 whole zebrafish embryos for each sample, and subjected to cDNA synthesis using ProtoScript M-MuLV First Strand cDNA Synthesis Kit (New England BioLabs). mRNA levels were examined by RT-PCR using *ef1a* as a control. Primers used in RT-PCR are:

*med12*-primer #1, sense, 5′-GCTCTGGTCTGGCACTACTC-3′, antisense, 5′-CTGTTGTCTCCTGACACTTG-3′; *med12*-primer #1, sense, 5′-CTAAGCTGCATGCTACAGAGTAT-3′, antisense, 5′-CCTTTGCCCG AACCTGTTG-3′; *nipbla*, sense, 5′-GGCTACATGCAGTACAGCCA-3′, antisense, 5′-CATCGTACGGGGTTCCACTA-3′; *nipblb*, sense, 5′-CAGACCCAGAAGGAGAGCT-3′, antisense, 5′-CTTGGTCCGAGTCGTCGTAT-3′; *ef1a*, sense, 5′-TCAGCGCATACATCAAGAAGA-3′, antisense, 5′-CTGTGCAGACTTTGTGACCT-3′.

The *med12*-primer #1 was designed to detect both precursor (including an intron of about 600 bases) and mature mRNA, whereas *med12*-primer #2 was designed at junctions of exons to detect only mature mRNA [98].

For Q-RT-PCR of mouse tissue, total RNA was isolated from somite-staged mouse hindlimbs from E10.5 embryos (WT *n* = 6, mutant *n* = 7) using the RNeasy minikit (QIAGEN). cDNA was synthesized from RNA using the iScript Reverse Transcription Supermix for RT-qPCR (*BioRad*). cDNA was PCR amplified using the iQ SYBR green Supermix (*BioRad*) with a CFX96 Real-Time System (*Bio-Rad*). Expression changes were normalized to beta-2 microglobulin, and the expression of each gene was calculated using the 2^−ΔΔCt^ method. A Student's t test was used for statistical analysis.

Primers:

*B2m* atgggaagccgaacatactg cagtctcagtgggggtgaat

*Nipbl* agtccatatgccccacagag accggcaacaataggacttg

*Shh* ggaactcacccccaattaca tcatcacagagatggccaag

*Ptch1* gccacagcccctaacaaaaat acccacaatcaactcctcctg

*Hand2* ccgacaccaaactctccaag tcttgtcgttgctgctcact

*Hoxa13* ctggaacggccaaatgtact cctataggagctggcgtctg

*Hoxc4* ccagcaagcaacccatagtc ctcagagaggcacagcgagt

*Hoxc6* ccaggaccagaaagccagta ccgagttaggtagcggttga

*Hoxc13* taccagcactgggctctttc gaatttgctggctgcgtact

*Hoxd4* ccctgggaaccactgttct ctccctgggctgagactgt

*Hoxd8* gaggccgagctggtacaata ctagggtttggaagcgactg

*Hoxd9* gctgaaggaggaggagaagc gcgtctggtatttggtgtagg

*Hoxd10* ggagcccactaaagtctccc tttccttctcctgcacttcg

*Hoxd11* aaagagcggcggcacagt aaagaaaaactcgcgttcca

*Hoxd12* aaggcaccaagtatgactacgc atctgctgctttgtgtagggt

*Hoxd13* tggaacagccaggtgtactg tggtgtaaggcacccttttc

### Cyclopamine (CyA) treatment

CyA was prepared at 10 mM in ethanol and stored at −20°C. Zebrafish embryos were incubated in CyA at 50 µM in embryo medium starting at 8 hpf in the dark and fixed with 4% paraformaldehyde (PFA) at indicated stages for ISH.

### Proliferation and cell death

Cell proliferation was examined by bromodeoxy uridine (BrdU) incorporation assay as previously reported [98]. Incorporated BrdU was detected by staining with a rat monoclonal anti-BrdU antibody (Abcam, 1∶100) and an anti-rat Alexa488-conjugated secondary antibody (Invitrogen, 1∶200). Nuclei of acid-treated samples were stained with DAPI (0.5 µg/mL). Levels of proliferation were quantified by calculating a ratio of BrdU-positive cells to DAPI-stained total cells. P-values were determined by student *t*-test

Cell death was examined by the terminal deoxynucleotidyl transferase-mediated dUTP nick end labeling (TUNEL) assay and acridine orange staining. For TUNEL assays, embryos were fixed at indicated stages with 2% PFA for 2 hr at room temperature and then washed in PBS containing 0.1% Triton-X-100. The fixed embryos were dehydrated in a graded series of methanol, permeabilized in cold-acetone for 10 min at −20°C, and then treated with proteinase K (10 µg/ml for 10 min at room temperature). Fragmented genomic DNA in dying cells was detected by using In Situ Cell Death Detection Kit (Roche). Dying cells were also detected by staining whole live embryos with acridine orange (5 µg/mL) for 5 min.

### Microarray analysis

Total RNA was extracted from hindlimbs (left and right) from each of 12 *Nipbl^+/+^* and 12 *Nipbl^+/−^* mouse embryos (E10.5, somite stages 35–38) [3]. The RNA was further processed by the UCI Genomics High-Throughput Facility for microarray analysis using Affymetrix Mouse Gene 1.0 ST arrays. The 24 probe cell intensity files (.Cel) were pre-processed using the Expression File Creator program of GenePattern (Broad Institute) and statistical analysis was performed using the Comparative Markers Selection module. Raw data will be made freely available to the public through Gene Expression Omnibus (http://www.ncbi.nlm.nih.gov/geo/query/acc.cgi?acc=GSE60932; accession number GSE60932).

### Three dimensional-fluorescence in situ hybridization (3D-FISH)

Zebrafish embryos were fixed at indicated stages in 4% PFA and sagittal cryosections cut at a thickness of 20 µm. FISH was performed on sections as described elsewhere [99]. Briefly, sections were permeabilized in 0.5% Triton X-100 in PBS and then genomic DNA was unmasked by 9 cycles of incubation at 90°C using a microwave and cooling for 2 min in 10 mM sodium citrate buffer (pH 6.0). Sections were then permeabilized in acetone for 5 min at −20°C and incubated in 50% formamide in 1× SSC for at least 4 hours. Pretreated sections were loaded with probe solution prepared in hybridization buffer (50% formamide, 10% dextran sulfate in 1× SSC), covered with a cover slip, sealed with rubber cement and prehybridized for at least 2 hr at 37°C. Probes were heat-denatured by incubating the slides at 80°C for 5 min, and hybridized at 37°C for 2–3 days. After washing in 0.1× SSC at 60°C, nuclei were stained with DAPI (0.05 µg/mL) and slides were mounted for fluorescence microscopy.

Fluorescent probes for FISH were labeled with dUTP conjugated with Alexa 488 or Alexa 568 (Invitrogen) using a Nick Translation Mix (Roche) according to the manufacturer's instructions and purified as described elsewhere [100]. For labeling, 1 µg of BAC DNA purchased from the BACPAC resource center was used as a template, and 250 ng each of the labeled probes per slide were used for hybridization. Zebrafish BAC clones used for *hoxd*, 3′, and 5′ probes are CH73-86I10, CH73-267A7, and CH73-381A1, respectively.

### Image analysis

Slides were examined using an Olympus confocal microscope (FV1000) and multiple optical sections along the z-axis were taken in 0.1 µm intervals. Captured images were analyzed using ImageJ. Outlines, areas, and central coordinates along x, y and z axes were measured for each fluorescent signal using the Wand tool in Image J in combination with ROI manager, and spatial distances between two closely located and differently colored signals were calculated. 60 nuclei from pectoral fin buds and 40–45 nuclei from hindbrain were analyzed for each embryo, and 3–7 embryos were used for each condition/probe set tested. Normalized inter-probe distances were plotted in probability histograms showing the mean percentage (± SD) of total nuclei from each sample displaying a given separation between fluorescent dots. Statistical significance was determined by the Mann-Whitney U test.

## Supporting Information

Figure S1 Expression of *nipbla* and *nipblb* in developing pectoral fin buds. (A) Expression of *nipbla* and *nipblb* in pectoral fin buds examined by ISH. Dorsal views, anterior to the top. (B) Transverse sections of pectoral fin buds at 36 hpf showing *nipbla* and *nipblb* expression in fin mesenchyme (me) rather than apical ectoderm (ec). Expression is also detected in endoderm (en) and neural tube (nt) but not somites (so).
(EPS)

Figure S2 Reduced pectoral fins in Nipbl-deficient embryos. Morphologies of live control (A, B, E, F) and Nipbl-deficient embryos (C, D, G, H) at 60 hpf (A–D) and 76 hpf (E–H). Dorsal (A, C, E, G) and lateral (B, D, F, H) views, anterior to the left. Pectoral fins are reduced in Nipbl-deficient embryos (arrows) and anterior ends of lower jaws are indicated (arrowheads).
(EPS)

Figure S3 Nipbla and Nipblb act cooperatively in pectoral fin development. Morphologies of live control (A), Nipbl-deficient (B) and embryos injected with either *nipbla*-MO (C, D) or *nipblb*-MO (E, F) at indicated amounts. Dorsal views, anterior to the left. (G) Whisker plots of fin length (medians): 431.8 µm, n = 20 (control), 259.1 µm, n = 40 (*nipbla/b*-MO), 385.3 µm, n = 40 (*nipbla*-MO, 0.75 ng), 351.0 µm, n = 40 (*nipbla*-MO, 1.5 ng), 417.6 µm, n = 40 (*nipblb*-MO, 0.75 ng), and 399.7 µm, n = 20 (*nipblb*-MO, 1.5 ng). *: p<10^−6^.
(EPS)

Figure S4 Small fin phenotypes could be caused by reduced proliferation rather than cell death of fin mesenchymal cells. (A, B) Cell death in pectoral fins of control (A) and Nipbl-deficient larvae (B) at 120 hpf was examined by TUNEL assay. (C–E) Cell death in pectoral fin buds of Nipbl-deficient at 40 hpf was examined by TUNEL assay in comparison with stage-matched (C, 36 hpf) and time-matched (D, 40 hpf) comparisons. TUNEL positive cells are shown in red. Lateral views with anterior to the left. (F–I) Proliferation of pectoral fin buds was examined by BrdU incorporation assay. Stage-matched (F, 36 hpf) and time-matched (G, 40 hpf) controls were compared with Nipbl-deficient embryos (H, 40 hpf). BrdU-positive cells (green) and DAPI stained cells (blue). Lateral views, anterior to the left. (I) Cell proliferation was quantitatively analyzed by determining proportions of BrdU-positive cells in fin mesenchymal cells stained with DAPI. Ave ± S.D.: 73.9±5.29% (n = 8, control at 36 hpf), 73.0±3.78% (n = 9, control at 40 hpf), 48.7±12.0% (n = 12, *nipbla/b*-MOs-injected embryos at 40 hpf). p-values compared with control at 36 hpf were determined by student *t*-test. *: p<0.001.
(EPS)

Figure S5 Staging embryos by posterior lateral line primordium position. (A) Posterior lateral line (pLL) primordia (red arrows) detected by ISH for *fgf10a* at indicated stages move progressively posterior relative to pectoral fin buds (yellow arrows) from 22–48 hpf, indicating a 3–4 hour and 6 hour developmental delay in Nipbl-deficient embryos at 36 hpf and 48 hpf, respectively. Duplicated signals at the tail tips in some panels reflect dorsoventral bifurcation of the tail, rather than ectopic expression of *fgf10a*, which is a typical phenotype observed in Nipbl-deficient embryos [11]. Lateral views. (B) Summary of stage-match comparisons between control and Nipbl-deficient embryos.
(EPS)

Figure S6 Rescue of AER-*fgf* gene expression in pectoral fin buds of Nipbl-deficient embryos by exogenous *nipbla* mRNA. Expression of the AER *fgf* genes, *fgf16* and *fgf8a*, in pectoral fin buds (arrows) of controls, embryos injected with *nipbla/b*-MOs, and those co-injected with *nipbla/b*-MO and 400 pg of *nipbla* mRNA was examined by ISH at 48 hpf. Dorsal views, anterior to the top.
(EPS)

Figure S7 Expression of genes in the *fgf10a* signaling pathways is unaffected in Nipbl-deficient embryos. Expression of *fgf10a* (A; arrows), *tbx5a* (B) and *fgf24* (C) in control and Nipbl-deficient embryos was examined by ISH at indicated stages. *fgf10a*-expressing pLL primordia are marked by asterisks. Dorsal views, anterior to the top.
(EPS)

Figure S8 Rescue of mesenchymal gene expression in pectoral fin buds of Nipbl-deficient embryos by exogenous *nipbla* mRNA. Expression of genes in the *shha*/*hand2*/5′-*hox* gene cassette at 36 hpf in pectoral fin buds of controls, embryos injected with *nipbla/b*-MOs, and those co-injected with *nipbla/b*-MO and 400 pg of *nipbla* mRNA was examined by ISH. Dorsal views, anterior to the top.
(EPS)

Figure S9 Expression of genes in the RA signaling pathways is unaffected in Nipbl-deficient embryos. Expression of the RA synthesizing enzyme *aldh1a2* and the RA-degrading enzyme *cyp26a1*, a target of the RA signaling, at 13 (upper) and 19 (lower) hpf in Nipbl-deficient embryos. Anterior somites are indicated by brackets. Dorsal views, anterior to the top.
(EPS)

Figure S10 Expression of *hox* genes along the anterior-posterior axis of the neural tube is unaffected in Nipbl-deficient embryos. *hox* gene expression at 36 hpf examined by ISH. Lateral views, anterior to the left. Pectoral fin buds are indicated by black arrows. Anterior and posterior limits of *hox* expression in the neural tube are indicated by red arrowheads.
(EPS)

Figure S11 Gene expression in pectoral fin buds of CyA-treated embryos. (A) Effects of CyA treatment on development was examined by A-P positioning of pLL primordial expressing *fgf10a*. Lateral views with anterior to the left. Pectoral fin buds (yellow arrows) and pLL primordial (red arrows) are pointed. Expression of *fgf10a* in pLL primordial and pectoral fin buds is reduced by CyA treatment and becomes undetectable in pLL primordia by 36 hpf. (B) Expression of mesenchymal genes in pectoral fin buds was examined by ISH in possible stage-match comparisons. Dorsal views, anterior to the top.
(EPS)

Figure S12 Analysis of expression of all mouse *Hox* genes in wildtype and *Nipbl* ^+/−^ hindlimb buds. (A–D) Expression values show hybridization intensity for probe sets representing all of genes in the four *Hox* clusters (although the relationship between hybridization intensity and transcript abundance is not necessarily the same for different probesets, intensity gives a rough sense of abundance). Data are graphed as mean ± SEM for the mutant (red) and wild type (blue) samples (see Experimental Procedures). Asterisks indicate genes also shown in Table 1, for which expression changes were observed with strong or moderate statistical significance (1%<FDR<7.5%; double asterisk) or weak statistical significance (FDR<25%; single asterisk); filled arrows show the directions in which expression changes were observed. Open arrows show directional changes that were also tested and confirmed by Q-RT-PCR (see Table 1), whereas “*n.c.*” marks genes that were tested by Q-RT-PCR and showed no detectable change in expression. The open arrow by *Hoxd8* is marked with a question mark because Q-RT-PCR showed a 27% elevation in expression in *Nipbl*+/− limb buds, but the result was not statistically significant (P = 0.14). The tight error bars on the microarray data for most transcripts, and the confirmation of all significant results by PCR, justify the ability to attach statistical significance even to these modest effect sizes. Overall, the data illustrate that expression changes in *Nipbl* ^+/−^ limb buds are biased toward the 5′ ends of the *Hox* clusters, and suggest that, as in zebrafish, effects occur at all of the expressed clusters (*HoxA*, *C* and *D*). In the *HoxD* cluster, which exhibits the highest overall hybridization intensities, the largest fold decreases are seen at the extreme 5′ end (*Hoxd12*, *Hoxd13*), and give way, as one moves in the 3′ direction, to a weak decrease (*Hoxd11*) and then a statistically significant increase (*Hoxd10*, and possibly *Hoxd8*). Thus, even though these effects are much smaller in magnitude than in zebrafish (where *nipbl* knockdown is likely much greater than in *Nipbl* ^+/−^ mice), they appear to show similar positional trends. (E–F) Whole mount in situ hybridization for *Hoxd12* (E) and *Hoxd13* (F) in E10.5 limb buds of *Nipbl^+/−^* and wildtype mice. For both *Hox* genes, expression is reduced significantly in both forelimbs and hindlimbs, consistent with the results of microarray analysis and Q-RT-PCR (panels A–D, and Table 1).
(EPS)

Figure S13 Expression of genes in the *fgf10a* pathways is unaffected in Med12-deficient embryos. Expression of *fgf24* (30, 48 hpf) and *fgf10a* (36 hpf) in Med12-deficient embryos examined by ISH. Dorsal views, anterior to the top.
(EPS)

Figure S14 Expression of *med12* in Nipbl-deficient embryos. Expression of *med12* in embryos injected with *nipbla/b*-MOs was examined by RT-PCR at 8 and 36 hpf. *ef1a* was used as a control.
(EPS)

Figure S15 Developmental delay of Rad21-deficient embryos. Effects of Rad21-deficiency (*rad21*-MO at 2.5 ng/embryo) on embryonic development were examined by A-P positions of pLL primordia by ISH for *fgf10a*. Lateral views, anterior to the left. Pectoral fin buds (yellow arrows) and pLL primordial (red arrows) are pointed. Expression of *fgf10a* in pLL primordial and pectoral fin buds of Rad21-deficient embryos were detected at levels significantly lower than control and lost by 54 hpf.
(EPS)

Figure S16 Expression of genes in pectoral fin buds of Rad21-deficient embryos. Effects of Rad21 reduction of expression of mesenchymal genes in pectoral fin buds were examined by ISH in possible stage-match comparisons. Dorsal views, anterior to the top.
(EPS)

Figure S17 Chromosome conformation around the *hoxda* locus in hindbrain. (A, B) Effects of Nipbl- and Med12-reduction on a higher-order chromatin conformation in hindbrain was examined by 3D-FISH at 38 hpf. Interprobe distances between (A) *hoxd* and 3′ probes and (B) *hoxd* and 5′ probes shown by Whisker plots. Details of medians, numbers of nuclei and embryos, and p-values are shown in Table 2. Dotted lines indicate thresholds for separated (upper) and closed (lower) signals in Table 2.
(EPS)
